# Making Gynogenetic Diploid Zebrafish by Early Pressure

**DOI:** 10.3791/1396

**Published:** 2009-06-30

**Authors:** Charline Walker, Greg S. Walsh, Cecilia Moens

**Affiliations:** Institute of Neuroscience, University of Oregon; Division of Basic Science, Fred Hutchinson Cancer Research Center - FHCRC

## Abstract

Heterozygosity in diploid eukaryotes often makes genetic studies cumbersome. Methods that produce viable homozygous diploid offspring directly from heterozygous females allow F1 mutagenized females to be screened directly for deleterious mutations in an accelerated forward genetic screen. Streisinger et al.^1,2^ described methods for making gynogenetic (homozygous) diploid zebrafish by activating zebrafish eggs with ultraviolet light-inactivated sperm and preventing either the second meiotic or the first zygotic cell division using physical treatments (heat or pressure) that deploymerize microtubules. The "early pressure" (EP) method blocks the meiosis II, which occurs shortly after fertilization. The EP method produces a high percentage of viable embryos that can develop to fertile adults of either sex. The method generates embryos that are homozygous at all loci except those that were separated from their centromere by recombination during meiosis I. Homozygous mutations are detected in EP clutches at between 50% for centromeric loci and less than 1% for telomeric loci. This method is reproduced verbatim from the Zebrafish Book^3^.

**Figure:**
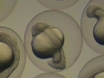


## Protocol

### Overview of early pressure

Note: recipes for many of the reagents used in this protocol such as tricaine, fish water and Hanks saline are available online in chapter 10 of the Zebrafish Book^3^ (http://zfin.org/zf_info/zfbook/zfbk.html)

Produce embryos by in vitro fertilization using UV inactivated sperm.
Immediately transfer the fertilized eggs to a glass vial (with a plastic snap cap) filled to the brim with egg water. Cover the open end of the vial with a thin sheet of latex rubber and snap the top (into which a hole has been cut) onto the vial over the rubber.
Place the vial in a hydraulic press and at 1.4 min after fertilization raise the pressure to 8,000 p.s.i. (Note that many presses are calibrated to read the pressure exerted on a 2-inch diameter piston, and readings must be converted to p.s.i.)
Beginning at 6 min after fertilization, gradually release the pressure over a period of 1 min.

### Detailed early pressure procedure

**Day before experiment**
  In the late afternoon, separate males and females to be squeezed. Any old male can be used since he will not be contributing genetic material. Females should look large and fat in the belly. Males should look yellow and spry.
  Hold females and males separately overnight in holding tanks where they can be easily accessed in the morning.
**Morning of experiment**
  Prepare Hanks solution; add sodium bicarbonate.
  Bring to fish facility: ice bucket, Hanks, Tricaine, timer, tubes for collecting sperm.
  Dilute Tricaine in fish water
**Collect sperm**
  Prepare a clear vial of Hanks solution on ice. Measure ~50 μl per male to be squeezed.
  Anesthetize 3 or 4 males at a time by immersion in tricaine. They can be lifted out of the anesthetic with a plastic spoon.
  Rinse in fish water and blot "damp-dry" on a paper towel (very important: water activates sperm).
  Mount fish in a foam holder under a stereomicroscope at low-magnification with epi-illumination. Separate the pelvic fins with forceps and position a microcapillary at the opening of the cloaca.
  Stroke the sides of the fish gently but firmly with smooth (Millipore) forceps.
  As the milky sperm come out of the genital pore, collect it in the microcapillary using gentle suction.
  Pool the sperm from several males in ice-cold Hank's saline. Sperm from 5-10 males is adequate for fertilization of several hundred eggs. Sperm in cold Hanks's will continue to fertilize eggs efficiently for several hours or even days. "Eyeball" the concentration of sperm, collecting enough to make a cloudy suspension.
**UV inactivate sperm**
  Fill the bottom of a glass Petri dish with ice.
  Put a watchglass on top of the ice in the dish.
  Using a Pasteur pipette, transfer the sperm from the test tube onto the watchglass. Be sure to get as much of the sperm into the pipette as possible without taking up any bubbles of air with it. Also, do not bubble the sperm in the watchglass as you are expelling them from the pipette. Expel sperm in a thin, even layer on watch glass. DISCARD THIS PIPETTE.
  Place the Petri dish into a UV cross-linker
  Turn on crosslinker for 2 minutes
  Using a CLEAN pipette, remove the sperm from the watchglass and transfer it to an eppendorf tube marked UV Sperm and place on ice.
**Egg collection and in vitro fertilization**
  Anesthetize three or four females at a time in tricaine.
  Rinse in fish water and blot "damp-dry" on a paper towel. Excess water will swell the eggs and prevent fertilization.
  Place the female in a 35 mm plastic petri dish and with damp (not wet) fingers, press gently but firmly on the belly. If she is prepared to lay eggs, they will come out quite easily.
  Gather the eggs with a spatula and return the female to water. Good eggs are a yellowish, translucent color, whereas eggs that have remained in the female too long are white and watery (see below). To ensure getting good eggs, collect them during the first 2 hours after the lights come on in the fish facility. Keep eggs covered to prevent drying-out.
  Add 30-50 μl of the UV sperm suspension to the eggs, stir gently with pipette tip.
  Immediately add about 1 ml of fish water *and start timer*. Eggs left to develop at this point will be haploids.
**Early Pressure. *Note: steps a-g must be accomplished within 90 seconds of fertilization!***
  Start timer at moment that eggs are activated by the addition of water
  Wait a few moments and add more water, swirl the eggs to the center of the dish.
  Using a cut-off and polished Pasteur pipette, transfer the fertilized eggs to the pressure vials.
  If needed, add more egg water to the vials so that they are almost overflowing, leaving a bead of water at the lip of the vials.
  Place a rubber top over the bead of water and secure the plastic lid over the rubber.
  Place the vial(s) upside down in the pressure cylinder, adding more egg water to refill the cylinder. Put the top on the cylinder securely.
  Place the cylinder in the press with the plunger up and apply 8,000 lbs/sq. in.
  Leave the cylinder in the press until 6 min after the time of activation (for a total time of 4.5 min of pressure).
  At 6 min after activation, remove the cylinder from the press, and remove the vials from the cylinder. Carefully dry the vials to prevent cooling of the embryos.
  Reset the cylinder and refill it with egg water.
  Prepare the next vials by partially filling them with egg water as needed.
  Repeat for all the batches of eggs.

NOTE: The cylinder will hold up to 3 vials at a time, so you may want to delay fertilization of eggs a few minutes to see if other females already in the tricaine give eggs. This will help speed things up by treating more batches at one time. Also, remember once the press is being used do not put any more fish in the tricaine until the treatment is completed. Allowing fish to stay in the anesthetic too long can kill them. Secondly, if you squeeze fish while the press is being used, and get eggs, they may be too dry to be viable by the time the press can be used again.

## Discussion

It is important to make sure that embryos produced by this method are true gynogenetic diploids. As controls, generate a clutch of normal diploid eggs (by keeping aside some of the sperm without UV-inactivation) and a clutch of haploid embryos (by keeping aside a clutch of eggs fertilized with UV sperm without going through the EP procedure). At 1 day post fertilization haploid embryos have a short body axis, irregular brain and somite morphology. Haploids are not viable after 2-3 days. EP diploids should look like normal diploids, although some irregular "EP monsters" may occur. Clutches of EP diploids should be largely (70-90%) viable. The presence of haploids in the EP diploid clutch suggests a failure in the EP process (for example, the press failing to hold pressure). The presence of diploids in the haploid clutch suggests incomplete sperm inactivation (for example a failure in the crosslinker). 

EP diploids have been used to determine gene-centromere distances^1^ to map mutants identified in forward genetic screens^4,6^. Forward screens of EP diploid embryos are an efficient way to identify genes involved in both early and later developmental processes and are being used today^5,7^.
